# Optimizing Initial Vancomycin Dosing in Hospitalized Patients Using Machine Learning Approach for Enhanced Therapeutic Outcomes: Algorithm Development and Validation Study

**DOI:** 10.2196/63983

**Published:** 2025-03-31

**Authors:** Heonyi Lee, Yi-Jun Kim, Jin-Hong Kim, Soo-Kyung Kim, Tae-Dong Jeong

**Affiliations:** 1 Interdisciplinary Program in Bioinformatics, College of Natural Sciences Seoul National University Seoul Republic of Korea; 2 Department of Environmental Medicine College of Medicine Ewha Womans University Seoul Republic of Korea; 3 Graduate Program in System Health Science and Engineering Ewha Womans University Seoul Republic of Korea; 4 Department of Biological Sciences College of Natural Sciences Seoul National University Seoul Republic of Korea; 5 Department of Laboratory Medicine College of Medicine Ewha Womans University Seoul Republic of Korea

**Keywords:** algorithm, machine learning, therapeutic drug monitoring, vancomycin, area under curve, pharmacokinetics, vancomycin dosing

## Abstract

**Background:**

Vancomycin is commonly dosed using standard weight–based methods before dose adjustments are made through therapeutic drug monitoring (TDM). However, variability in initial dosing can lead to suboptimal therapeutic outcomes. A predictive model that personalizes initial dosing based on patient-specific pharmacokinetic factors prior to administration may enhance target attainment and minimize the need for subsequent dose adjustments.

**Objective:**

This study aimed to develop and evaluate a machine learning (ML)–based algorithm to predict whether an initial vancomycin dose falls within the therapeutic range of the 24-hour area under the curve to minimum inhibitory concentration, thereby optimizing the initial vancomycin dosage.

**Methods:**

A retrospective cohort study was conducted using hospitalized patients who received intravenous vancomycin and underwent pharmacokinetic TDM consultation (n=415). The cohort was randomly divided into training and testing datasets in a 7:3 ratio, and multiple ML techniques were used to develop an algorithm for optimizing initial vancomycin dosing. The optimal algorithm, referred to as the OPTIVAN algorithm, was selected and validated using an external cohort (n=268). We evaluated the performance of 4 ML models: gradient boosting machine, random forest (RF), support vector machine (SVM), and eXtreme gradient boosting (XGB). Additionally, a web-based clinical support tool was developed to facilitate real-time vancomycin TDM application in clinical practice.

**Results:**

The SVM algorithm demonstrated the best predictive performance, achieving an area under the receiver operating characteristic curve (AUROC) of 0.832 (95% CI 0.753-0.900) for the training dataset and 0.720 (95% CI 0.654-0.783) for the external validation dataset. The gradient boosting machine followed closely with AUROC scores of 0.802 (95% CI 0.667-0.857) for the training dataset and 0.689 (95% CI 0.596-0.733) for the validation dataset. In contrast, both XGB and RF exhibited relatively lower performance. XGB achieved AUROC values of 0.769 (95% CI 0.671-0.853) for the training set and 0.707 (95% CI 0.644-0.772) for the validation set, while RF recorded AUROC scores of 0.759 (95% CI 0.656-0.846) for the test dataset and 0.693 (95% CI 0.625-0.757) for the external validation set. The SVM model incorporated 7 covariates: age, BMI, glucose, blood urea nitrogen, estimated glomerular filtration rate, hematocrit, and daily dose per body weight. Subgroup analyses demonstrated consistent performance across different patient categories, such as renal function, sex, and BMI. A web-based TDM analysis tool was developed using the OPTIVAN algorithm.

**Conclusions:**

The OPTIVAN algorithm represents a significant advancement in personalized initial vancomycin dosing, addressing the limitations of current TDM practices. By optimizing the initial dose, this algorithm may reduce the need for subsequent dosage adjustments. The algorithm’s web-based app is easy to use, making it a practical tool for clinicians. This study highlights the potential of ML to enhance the effectiveness of vancomycin treatment.

## Introduction

Vancomycin is a first-line treatment for methicillin-resistant *Staphylococcus aureus* (MRSA) infections [1,2]. Due to its narrow therapeutic index, therapeutic drug monitoring (TDM) of vancomycin is essential to achieving effective outcomes while minimizing side effects such as nephrotoxicity and ototoxicity [3-6]. The first consensus vancomycin TDM guidelines recommended area under the curve over 24 hours to minimum inhibitory concentration (AUC24/MIC) as the preferred pharmacokinetic/pharmacodynamic parameter [7]. However, calculating AUC24/MIC requires multiple blood draws which limits its routine clinical use. To simplify monitoring, trough serum vancomycin concentrations (15-20 mg/L) were previously recommended as a surrogate marker for AUC24/MIC, but the correlation between vancomycin’s AUC24/MIC and trough concentration remains modest [7,8]. Recently, with the increasing availability of Bayesian modeling software, the revised vancomycin TDM guidelines now advocate for AUC24/MIC-guided dosing (targeting 400-600) instead of trough-guided monitoring [6,9]. If AUC24/MIC is below 400, therapeutic efficacy is inadequate, necessitating a dose increase. Conversely, exceeding 600 suggests an increased toxicity risk, requiring dose reduction. A study by Tsutsuura et al [10] has demonstrated that AUC-guided dosing reduces acute kidney injury compared with trough-guided monitoring.

Despite these advances, determining the appropriate initial vancomycin dose remains challenging due to high inter- and intraindividual PK variability, particularly related to body weight and renal function [11]. In a study by Yoon et al [12], 69.8% (2051/2570) of cases had initial vancomycin trough concentrations outside the therapeutic range (10-20 mg/L). According to the latest clinical guidelines on TDM for vancomycin, the recommended dosage for serious MRSA infections is 15-20 mg/kg every 8-12 hours [6]. While the vancomycin package insert provides renal function-based dosing guidelines, in real-world clinical practice, initial doses are often determined based on the clinician’s experience [13,14]. Suboptimal initial dosing may lead to therapeutic failure or toxicity, prolonging treatment duration. Therefore, optimizing the initial vancomycin dosing to account for patient-specific PK variables is critical [15].

Conventional vancomycin TDM requires serum drug concentration measurements 24-48 hours postadministration, followed by manual TDM analysis. These processes may take at least several days to obtain the formal TDM reports after the first vancomycin dose. Moreover, differences in analytical methods, reagents, and instruments across clinical laboratories can introduce variability in vancomycin measurements [16]. Given these challenges, an alternative approach that leverages machine learning (ML) to predict vancomycin AUC24/MIC before administration could significantly enhance dosing accuracy, enabling more precise dosing and patient-specific therapy.

Advancements in artificial intelligence and ML enable data-driven decision-making, improving personalized medicine across various medical fields [17-21]. By integrating ML into clinical practice, health care providers can make more informed decisions, optimize drug dosing, and minimize the time required to adjust treatments, ultimately leading to improved patient outcomes and greater resource efficiency. In the context of vancomycin dosing, an ML-driven approach could significantly enhance the precision of initial dosing, reducing the risk of under or overdosing and improving overall therapeutic success. To date, research on optimizing the initial vancomycin dosing using ML techniques with patient-specific PK variables and AUC24/MIC remains limited.

This study aimed to develop and evaluate an ML-based predictive algorithm for optimizing initial vancomycin dosing in hospitalized patients. By leveraging readily available clinical data and routine laboratory test results, our model predicts whether a given initial vancomycin dose achieves the therapeutic AUC24/MIC range (400-600). To enhance clinical accessibility, we also developed a web-based vancomycin TDM application to facilitate real-time implementation in clinical practice.

## Methods

### Study Design

**Figure 1:**
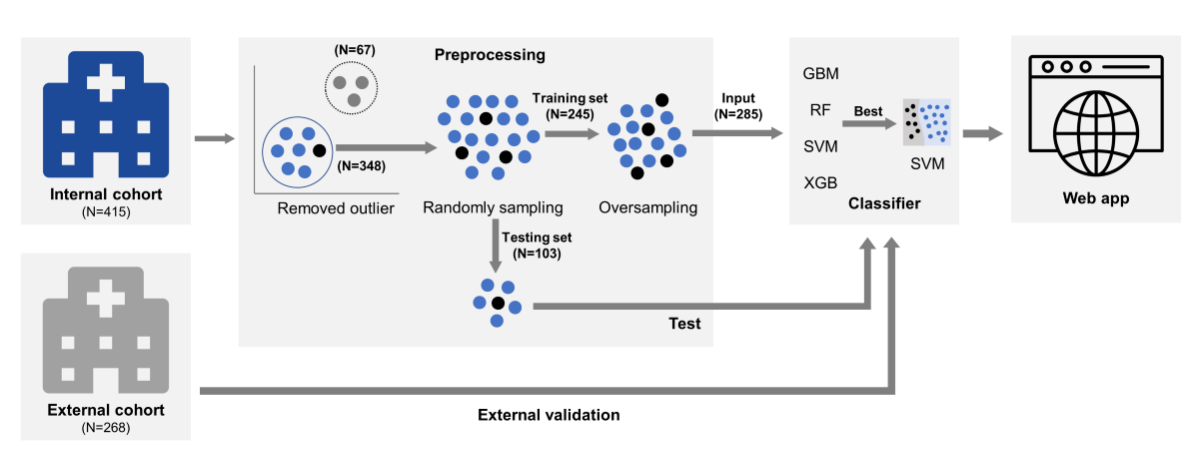
Study design. GBM: gradient boosting machine; RF: random forest; SVM: support vector machine; XGB: eXtreme gradient boosting.

This was a retrospective cohort study. The purpose of this study was to develop an algorithm using ML techniques to forecast whether the initial vancomycin regimen to be administered can achieve an AUC24/MIC ratio within the therapeutic range. In other words, the final output of the ML algorithm predicted “yes” or “no” based on whether the AUC24/MIC of vancomycin falls within the therapeutic range of 400 to 600. We established an internal cohort of patients who were administered intravenous vancomycin and underwent a TDM consultation to monitor the therapeutic dosage of vancomycin. Patients were treated at the discretion of the clinicians and not according to a standardized protocol. The internal cohort was randomly divided into training and testing datasets in a 7:3 ratio, and multiple ML techniques were used to develop an algorithm for determining the dosage for initial vancomycin administration. Then, the optimal algorithm was selected from the developed ones (hereinafter referred to as “OPTIVAN algorithm”), and its performance was validated using an external cohort. To enhance the clinical utility of the OPTIVAN algorithm, we implemented a web-based vancomycin TDM application (Figure 1).

### Internal Cohort

A total of 2770 cases from 1333 patients were referred for vancomycin TDM consultation to the Department of Laboratory Medicine at Ewha Womans University Seoul Hospital (Seoul, Republic of Korea) from August 2021 to September 2022. Of these, we selected the first TDM consultation data (n=540) in the same patients if multiple were requested, and excluded patients aged 18 years or younger (n=22), those undergoing hemodialysis (n=38) or estimated glomerular filtration rate (eGFR) less than 15 mL/min/1.73 m^2^ (n=10), patients with total medication time of less than 48 hours (n=43), patients with less than 3 medication doses (n=6), patients with vancomycin trough concentrations below the lower limit of quantification (n=4), patients with no trough measurement (n=1), and patients with serum creatinine concentration below the limit of quantification (n=1). These resulted in an internal cohort of 415 patients as a development dataset (Figure S1 in Multimedia Appendix 1).

### External Cohort

Patient data for external validation of the OPTIVAN algorithm was collected from Ewha Womans University Mokdong Hospital (Seoul, Republic of Korea) from March to September 2022. Among the 693 patients who underwent TDM consultation during the study period, 317 patients remained after removing duplicate records. We further excluded patients aged 18 years or younger (n=5), those undergoing hemodialysis (n=12), those without serum creatinine measurements (n=17), and patients with a total medication time of less than 48 hours (n=15). Consequently, a total of 268 patients were included as a validation dataset (Figure S1 in Multimedia Appendix 1).

### Feature Collection

Patients’ clinical information including demographics, vancomycin drug administration history, sampling time for drug concentration measurements, renal replacement therapy history, and vancomycin TDM consultation analysis data were reviewed. The following patient’s clinical information was collected as candidate features of input data to be used in algorithm development: age, sex, department, height, weight, BMI, body surface area, length of hospitalization, and main diagnosis (7 categories: bloodstream infection or sepsis, osteomyelitis, central nervous system infection, pneumonia, intra-abdominal infection, skin and soft tissue infection, and urinary tract infection); vancomycin drug-related information: blood drug concentration measurements (trough and peak), drug administration time, single drug dose, drug administration interval, number of drug doses, total drug administration time period, drug dose administered during the day, and daily drug dose per body weight; and laboratory data: white blood cell, red blood cell, hemoglobin, hematocrit, absolute neutrophil count, differential count (neutrophil, lymphocyte, and monocyte), total calcium, phosphorus, glucose, blood urea nitrogen (BUN), total protein, albumin, total bilirubin, alkaline phosphatase, aspartate aminotransferase, alanine aminotransferase, creatinine, eGFR, sodium, potassium, chloride, total carbon dioxide, high sensitive C-reactive protein, and procalcitonin.

All vancomycin TDM consultation analyses were performed based on AUC24/MIC-guided dosing using the Bayesian software, MwPharm++ (v.1.9.0.338; Mediware). We used the built-in 2-compartment model (model name: #vancomycin_adult_k_C2) with the population PK parameters for adults based on the Dutch Association of Hospital Pharmacists monograph [22,23]. In the vancomycin TDM analysis, the MIC was assumed to be 1 mg/L. The label value used to train the algorithm was the AUC24/MIC (acceptable if between 400 and 600, and unacceptable otherwise) when the eGFR was calculated by the 2009 creatinine-based chronic kidney disease epidemiology collaboration (CKD-EPI) equation [24,25]. In the Korean population, the 2009 CKD-EPI equation more accurately assesses renal function than the Cockcroft-Gault or 2021 CKD-EPI equations [26,27]. Since there was no clinically significant difference in the choice of eGFR equation used to calculate vancomycin’s AUC24 through Bayesian methods [23,25], we used the 2009 CKD-EPI equation to calculate eGFR.

### Data Preprocessing

After dividing the variables into categorical and continuous features, we performed imputation on the null values of continuous features. No imputation was performed for the categorical features, as these variables contained no null values. Imputation on the null values of noncategorical features was performed using “pmm” method in the R library “mice.” Outliers of the continuous features were defined as a value with an absolute value of the z score is 3.5 or more. The internal cohort was randomly divided into a 70% training set and a 30% testing set. To improve the quality of the training set for algorithm training, we excluded cases that were outliers from the continuous features and oversampled the training set. Oversampling was performed using “mwmote” method in the R library “imbalance.”

### Feature Selection

To ensure an unbiased evaluation of model performance, we used a bootstrapping methodology to derive the average performance metrics from 50 iterations. In each iteration, the internal cohort data was randomly partitioned in a 7:3 ratio into training and test sets. The model was trained on the training subset and subsequently evaluated on the test subset. By performing 50 iterations of bootstrapping throughout the entire process of deriving model performance, this iterative resampling process provides a robust estimate of the model’s generalization performance by mitigating the variability inherent in any single train-test split.

To develop an OPTIVAN algorithm that delivers the best performance while using the minimum number of features from our dataset, we selected the features to be used as input data. First, features that were easily obtainable, regardless of the hospital’s context, thereby ensuring practicality in data collection, were selected. A random forest (RF) algorithm was used for further feature selection based on the algorithm’s performance. The feature importance score of each feature in predicting vancomycin dose acceptability was calculated and ranked. The highest-ranked feature was then selected as the initial input for algorithm development, and the algorithm’s predictive power, as determined by the acceptability of AUC24/MIC of vancomycin, was assessed. Subsequently, we systematically evaluated the algorithm’s performance by incrementally introducing the next highest-ranked features. If the predictive performance increased and its predictive power improved, the additional feature was incorporated into the input data. Conversely, if there was no improvement in the predictive performance, the feature was not included. This sequential process continued until we reached a point where the model’s performance improvement plateaued with each additional feature. At that juncture, we determined the minimum number of features required for the optimal input data configuration. Finally, correlation analysis confirmed that the selected features were not highly correlated (Figure S2 in Multimedia Appendix 1).

### Feature Importance Calculation

To calculate the feature importance of our final model, which is based on the RF algorithm, the varImp() function from the varImp package was used. This function specializes in determining importance scores for RF models through a permutation-based method. Specifically, the prediction accuracy on the out-of-bag samples was recorded as reference data, and the data for each feature was permuted individually, recording the resulting prediction accuracy. By comparing the prediction accuracies of the reference data and the permuted data, the differences in accuracy were calculated. These differences were averaged over all trees and normalized by the SE. Features causing a greater decrease in prediction accuracy when permuted were assigned higher importance scores, indicating their significant role in the model’s predictive power.

### Development of the OPTIVAN Algorithm

We compared the prediction performance of different ML algorithms using selected features as input data. The ML algorithms evaluated were RF, gradient boosting machine, eXtreme gradient boosting (XGB), and support vector machine (SVM). We optimized the model parameters to achieve the best performance by using a grid search strategy for each algorithm. This involved defining a specific range for each parameter using the expand.grid() function from the caret package and using the tuneGrid option within the train function. This systematic approach allowed for an exhaustive search across specified parameter values to identify the optimal settings for our model. All algorithms were trained by applying 50 times bootstrap sampling to mitigate overfitting and ensure that the model’s performance is evaluated across different subsets of the data.

The performance of each algorithm model was evaluated by measuring the AUC of the receiver operator characteristic (AUROC) curve and AUC of the precision-recall curve (AUPRC), and the agreement between the model’s predicted probability and observed true probability in the calibration plot. In the calibration plot of each algorithm, the lower the sum of the absolute value of the difference between the algorithm’s predicted probability and the observed true predicted probability corresponding to each bin midpoint, the better the algorithm was considered to be with fewer under or overestimates. To ensure the robustness and reliability of our results, we conducted a resampling procedure. Specifically, for each algorithm, we resampled the 70% training dataset a total of 50 times to calculate the average AUROC and AUPRC values, and generate the calibration plot for a model with the highest AUROC and AUPRC values. The algorithm, which showed the highest average AUROC and AUPRC values, along with an optimal calibration plot, was selected as our ultimate model of choice, namely OPTIVAN algorithm. All statistical and computational analyses were carried out in R (version 4.2.3; R Development Core Team).

### External Validation and Subgroup Analyses

We conducted external validation and subgroup analyses to evaluate the performance of the OPTIVAN algorithm using an external cohort. Patients were stratified into subgroups based on their renal function (eGFR <60, 60-89, and ≥90 mL/min/1.73 m^2^), sex (male and female), and BMI (<18.5, 18.5-24.9, 25.0-29.9, and ≥30.0 kg/m^2^), and the performance of the OPTIVAN algorithm was evaluated within each subgroup.

### Implementing a Web-Based App Using the OPTIVAN Algorithm

**Figure 2:**
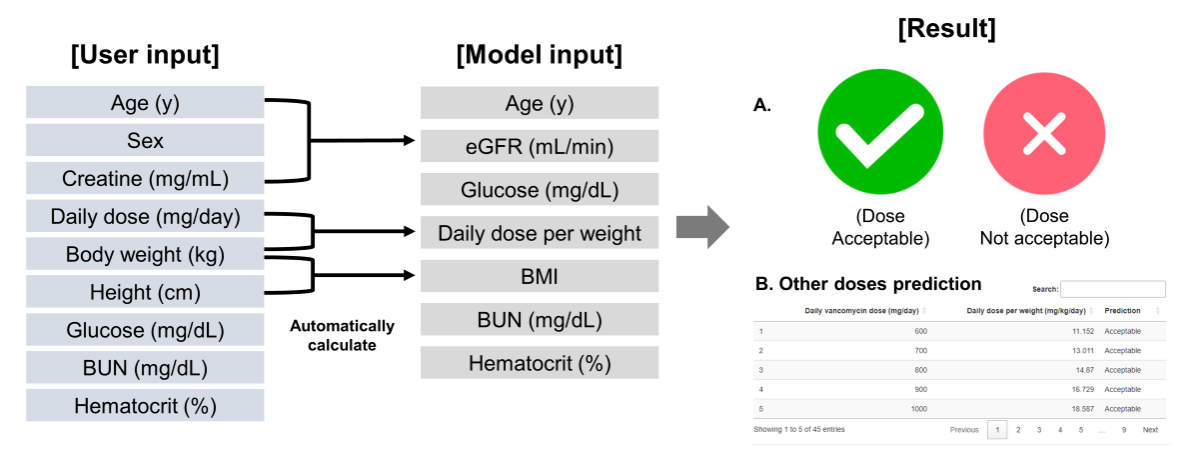
Summary of web-based app use. The user can predict whether the area under the curve over 24 hours to minimum inhibitory concentration (AUC24/MIC) of the initial vancomycin dosage they plan to administer falls within the therapeutic range. Upon entering the patient’s sex, intended initial dosage, height, weight, BUN, creatinine, glucose, and hematocrit results, the system automatically calculates the BMI, eGFR, and daily dose per body weight. It then promptly indicates whether the AUC24/MIC of vancomycin falls within the therapeutic range. Additionally, it provides examples of different drug dosages that fall within the therapeutic range. If the input dose value is unacceptable, the user can adjust the value and check again or view other acceptable doses in a table format. eGFR: estimated glomerular filtration rate.

The web-based app [28], was developed using Rshiny (version 1.7.4; RStudio). When a clinician enters the patient’s clinical information and laboratory test results (including sex, age, weight, height, BUN, creatinine, glucose, and hematocrit) and the desired daily dose of vancomycin for prescription, the system automatically calculates the BMI, eGFR in mL/min, and daily dose per body weight. Then, the algorithm quickly assesses whether the predicted AUC24/MIC of vancomycin falls within the therapeutic range. If the predicted AUC24/MIC falls within the therapeutic range, the clinician can proceed with administering the planned dose. If the predicted AUC24/MIC of vancomycin falls outside the therapeutic range based on the entered information, the app will notify the user that the drug dosage is inappropriate. The tool also offers easy-to-use tabular information. This table provides various daily dosage examples for achieving a therapeutic range of AUC24/MIC based on individual patient data. The description of the web-based app use is summarized in Figure 2, and an example of the screenshot is provided in Figure S3 in Multimedia Appendix 1.

### Ethical Considerations

This study was approved by the institutional review board of the Ewha Womans University Seoul Hospital (approval number SEUMC 2022-03-019-003) and Ewha Womans University Mokdong Hospital (approval number EUMC 2023-04-020). In light of the retrospective nature of the study, the process of obtaining informed consent from patients was exempted by both institutional review boards.

## Results

### Cohort Characteristics

The internal cohort of 415 patients included in the study had a mean age of 67.7 years and an average body weight of 61.9 kg. The most common reason for vancomycin administration was bloodstream infection or sepsis, affecting 245 (59.0%) patients, followed by skin and soft tissue infections in 59 (14.2%) patients. The mean eGFR was 90.9, with 66 patients exhibiting an eGFR of less than 60. The external cohort comprised 268 patients with a mean age of 69.9 years. Similar to the internal cohort, the predominant reason for vancomycin administration in this group was bloodstream infection or sepsis, which occurred in 154 (57.5%) patients. The mean eGFR of the external cohort was 82.8, significantly lower than that of the internal cohort. The detailed characteristics of the internal and external cohorts are summarized in Table 1.

**Table 1:** Baseline characteristics of internal and external cohorts.

Characteristics				Internal cohort (n=415)		External cohort (n=268)		*P* value	
Age (years), mean (SD)				67.7 (15.2)		69.9 (14.5)		.06	
**Sex, n (%)**								.80	
	Female			168 (40.5)		112 (41.8)			
	Male			247 (59.5)		156 (58.2)			
Height (meters), mean (SD)				163.2 (9.4)		162.9 (9.0)		.69	
Body weight (kg), mean (SD)				61.9 (13.6)		61.2 (13.0)		.52	
BMI (kg/m^2^), mean (SD)				23.2 (4.5)		23.0 (4.1)		.54	
**BMI group (kg/m^2^), n (%)**								.47	
	<18.5			56 (13.5)		35 (13.1)			
	18.5-24.9			226 (54.5)		158 (59.0)			
	25.0-29.9			107 (25.8)		63 (23.5)			
	≥30.0			26 (6.3)		12 (4.5)			
BSA^a^ (m^2^), mean (SD)				1.7 (0.2)		1.7 (0.2)		.56	
**Diagnosis, n (%)**								.08	
	Bloodstream infection or sepsis			245 (59.0)		154 (57.5)			
	Osteomyelitis			9 (2.2)		2 (0.7)			
	CNS^b^ infection			16 (3.9)		14 (5.2)			
	Pneumonia			41 (9.9)		43 (16.0)			
	Intra-abdominal infection			38 (9.2)		14 (5.2)			
	Skin and soft tissue infection			59 (14.2)		37 (13.8)			
	Urinary tract infection			7 (1.7)		4 (1.5)			
Hospitalization period (day), mean (SD)				39.9 (40.3)		35.4 (22.3)		.06	
**Vancomycin drug history, mean (SD)**									
	Dose (mg)			874.5 (193.7)		933.8 (180.5)		<.001	
	Dosing interval (h)			15.2 (5.6)		15.5 (5.8)		.58	
	Total number of administration			5.8 (2.3)		6.4 (3.4)		.008	
	Total drug administration time (h)			82.1 (28.1)		89.2 (39.4)		.01	
	Daily dose (mg/day)			1533.7 (535.5)		1623.9 (584.7)		.04	
	Daily dose per body weight (mg/day/kg)			25.7 (10.2)		27.4 (10.7)		.04	
	Trough concentration (mg/L)			10.4 (7.3)		12.9 (9.0)		<.001	
	AUC24/MIC^c^ ratio			472.9 (235.1)		531.4 (287.6)		.006	
	**AUC24/MIC ratio group, n (%)**								.06
	400-600			154 (37.1)		80 (29.9)			
	<400 or >600			261 (62.9)		188 (70.1)			
**Laboratory findings**									
	WBC^d^ (×10^9^/L), mean (SD)			9.5 (5.5)		10.8 (8.3)		.03	
	**Differential counts, n (%)**								
		Neutrophil	73.2 (17.3)		74.6 (13.9)		.25		
		Lymphocyte	16.2 (14.9)		14.9 (11.2)		.22		
		Monocyte	7.7 (4.1)		7.9 (3.9)		.53		
	ANC^e^ (×10^9^/L), mean (SD)			7.5 (5.2)		8.5 (7.6)		.06	
	RBC^f^ (×10^12^/L), mean (SD)			3.2 (0.6)		3.0 (0.7)		.02	
	Hemoglobin (g/dL), mean (SD)			9.7 (1.8)		8.9 (1.8)		<.001	
	Hematocrit, n (%)			29.0 (5.5)		27.0 (5.4)		<.001	
	Albumin (g/dL), mean (SD)			2.9 (0.5)		2.9 (0.6)		.53	
	ALP^g^ (IU/L), mean (SD)			127.0 (118.5)		129.2 (104.9)		.82	
	ALT^h^ (IU/L), mean (SD)			49.0 (120.0)		49.5 (123.3)		.96	
	AST^i^ (IU/L), mean (SD)			79.1 (361.4)		53.8 (111.6)		.19	
	Blood urea nitrogen (mg/dL), mean (SD)			22.1 (16.9)		24.3 (19.3)		.14	
	Chloride (mmol/L), mean (SD)			104.7 (5.4)		104.0 (5.6)		.15	
	Creatinine (mg/dL), mean (SD)			0.80 (0.52)		0.94 (0.74)		.008	
	eGFR^j^ (mL/min), mean (SD)			87.0 (30.4)		79.2 (31.1)		.001	
	eGFR (mL/min/1.73m^2^), mean (SD)			90.9 (29.5)		82.8 (29.6)		.001	
	**eGFR group (mL/min/1.73m^2^), n (%)**								.002
		< 60	66 (15.9)		65 (24.3)				
		60-89	242 (58.3)		122 (45.5)				
		≥90	107 (25.8)		81 (30.2)				
	Glucose (mg/dL), mean (SD)			138.8 (49.0)		136.7 (63.1)		.65	
	hsCRP^k^ (mg/dL), mean (SD)			7.3 (7.0)		9.1 (8.1)		.003	
	Phosphorus (mg/dL), mean (SD)			3.0 (0.8)		3.2 (0.9)		.002	
	Potassium (mmol/L), mean (SD)			3.9 (0.5)		4.0 (0.7)		.002	
	Procalcitonin (ng/mL), mean (SD)			3.5 (9.2)		0.9 (1.4)		.001	
	Sodium (mmol/L), mean (SD)			138.0 (5.5)		138.9 (5.5)		.03	
	TCO_2_^l^ (mmol/L), mean (SD)			25.4 (4.2)		22.9 (3.9)		<.001	

^a^BSA: body surface area.

^b^CNS: central nervous system.

^c^AUC24/MIC: area under the curve over 24 hours to minimum inhibitory concentration.

^d^WBC: white blood cell.

^e^ANC: absolute neutrophil count.

^f^RBC: red blood cell.

^g^ALP: alkaline phosphatase.

^h^ALT: alanine aminotransferase.

^i^AST: aspartate aminotransferase.

^j^eGFR: estimated glomerular filtration rate.

^k^hsCRP: high-sensitivity C-reactive protein.

^l^TCO_2_: total carbon dioxide.

### Feature Selection

In the process of feature selection for input data, we initially calculated the feature importance scores for each feature (Table 2). Among these features, age showed the highest feature importance score. Subsequently, we proceeded to identify features with notable importance scores that contributed to an enhancement in the algorithm’s AUROC when combined with age. This selection process led to the final choice of 7 features: age, eGFR (mL/min), daily dose per body weight, glucose, BUN, hematocrit, and BMI.

**Table 2:** Features filtered by feature importance score.

Feature	Importance score	SE
Age (years)	100	9.388×10^–4^
eGFR^a^ (mL/min)	74.03	7.243×10^–4^
Daily dose per body weight	70.96	8.626×10^–4^
Glucose	52.72	4.790×10^–4^
Height	45.54	4.905×10^–4^
ALT^b^	44.19	3.799×10^–4^
eGFR (mL/min/1.73 m^2^)	36.06	4.993×10^–4^
Chloride	35.85	4.395×10^–4^
Potassium	35.79	4.710×10^–4^
ALP^c^	35.77	3.717×10^–4^
Sodium	35.51	3.964×10^–4^
AST^d^	32.60	3.583×10^–4^
Blood urea nitrogen	32.40	4.128×10^–4^
hsCRP^e^	31.53	4.083×10^–4^
ANC^f^	30.56	3.202×10^–4^
TCO_2_^g^	30.46	3.777×10^–4^
Hematocrit	28.70	3.345×10^–4^
BMI	28.35	3.168×10^–4^
WBC^h^	27.24	3.459×10^–4^
Creatinine	25.24	3.420×10^–4^
Body weight	23.55	3.295×10^–4^
Daily dose	23.07	4.888×10^–4^
Total calcium	20.85	4.475×10^–4^
BSA^i^	19.27	3.101×10^–4^
Neutrophil	18.15	2.719×10^–4^
Lymphocyte	17.16	2.663×10^–4^
Monocyte	16.82	2.473×10^–4^
Total protein	16.18	3.218×10^–4^
Phosphorus	15.73	3.068×10^–4^
Total drug administration time	12.48	2.259×10^–4^
Dose	12.40	2.876×10^–4^
Hemoglobin	12.08	2.097×10^–4^

^a^eGFR: estimated glomerular filtration rate.

^b^ALT: alanine aminotransferase.

^c^ALP: alkaline phosphatase.

^d^AST: aspartate aminotransferase.

^e^hsCRP: high-sensitivity C-reactive protein.

^f^ANC: absolute neutrophil count.

^g^TCO_2_: total carbon dioxide.

^h^WBC: white blood cell.

^i^BSA: body surface area.

### Development of the OPTIVAN Algorithm

**Figure 3:**
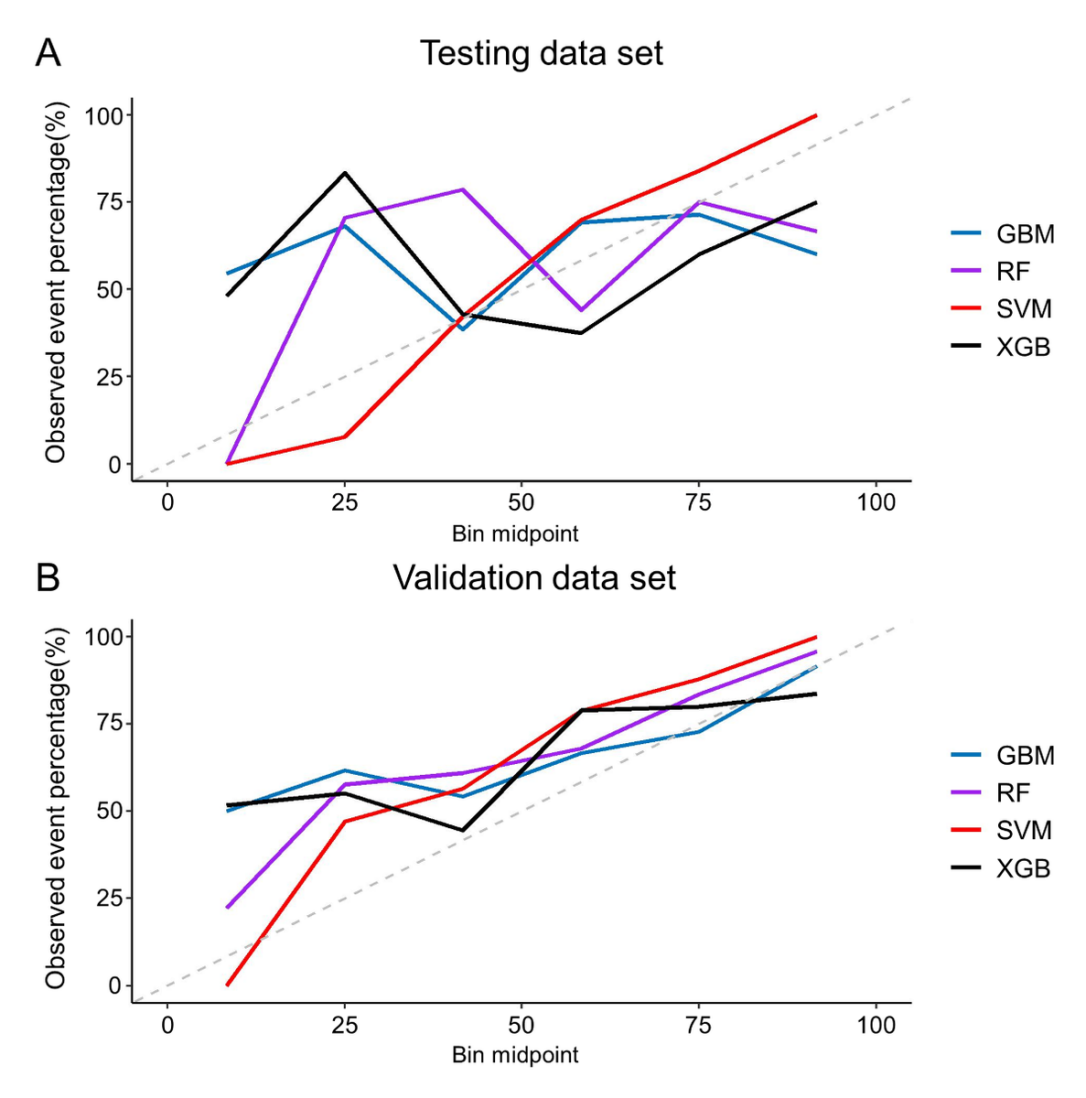
Calibration plots to compare algorithm. (A) The result of the testing dataset which is 30% of the internal cohort and (B) the result of the external validation dataset. All plots are based on the best result. Abbreviations: GBM: gradient boosting machine; RF: random forest; SVM: support vector machine; XGB: eXtreme gradient boosting.

The calibration plots for each ML algorithm are described in Figure 3. The SVM algorithm showed the highest AUROC (0.832 for the training dataset), AUPRC (0.817 for the training dataset), while other algorithms including the gradient boosting machine showed the lower AUROC (0.802 for the training dataset), AUPRC (0.729 for the training dataset; Table 3), and the most minimal discrepancy between the algorithm’s predictions and the actual observations in the calibration plot. Consequently, the model developed using the SVM algorithm was considered the most outstanding model and selected as the OPTIVAN algorithm. This model is a nonlinear model with radial basis function kernel, and tuned hyperparameters “cost” and “sigma,” each with a value of 1 and 0.3, respectively (the grid search range for both parameters was from 0.1 to 10, with a step size of 0.5).

**Table 3:** The predictive performance of machine-learning algorithms.

Method and dataset		Best performance						Average performance^a^				
		Sensitivity (95% CI)	Specificity (95% CI)	AUROC^b^ (95% CI)	AUPRC^c^ (95% CI)	*F*_1_-score	Sensitivity		Specificity	AUROC	AUPRC	*F*_1_-score
**GBM^d^**												
	Testing	0.730 (0.553-0.794)	0.725 (0.561-0.854)	0.802 (0.667-0.857)	0.729 (0.621-0.848)	0.767	0.727		0.542	0.711	0.758	0.720
	External validation	0.707 (0.637-0.771)	0.550 (0.435-0.662)	0.689 (0.596-0.733)	0.727 (0.662-0.797)	0.745	0.668		0.754	0.679	0.701	0.722
**RF^e^**												
	Testing	0.746 (0.621-0.847)	0.55 (0.385-0.707)	0.759 (0.656-0.846)	0.669 (0.549-0.800)	0.734	0.719		0.536	0.689	0.764	0.713
	External validation	0.702 (0.631-0.766)	0.538 (0.422-0.650)	0.693 (0.625-0.757)	0.702 (0.620-0.783)	0.739	0.682		0.584	0.695	0.701	0.733
**SVM^f^**												
	Testing	0.810 (0.691-0.898)	0.675 (0.509-0.814)	0.832 (0.753-0.900)	0.817 (0.738-0.916)	0.803	0.729		0.587	0.733	0.721	0.731
	External validation	0.660 (0.587-0.727)	0.688 (0.574-0.787)	0.720 (0.654-0.783)	0.672 (0.597-0.750)	0.736	0.674		0.636	0.720	0.631	0.737
**XGB^g^**												
	Testing	0.746 (0.621-0.847)	0.600 (0.433-0.751)	0.769 (0.671-0.853)	0.678 (0.562-0.803)	0.746	0.708		0.533	0.688	0.737	0.705
	External validation	0.707 (0.637-0.771)	0.650 (0.535-0.753)	0.707 (0.644-0.772)	0.697 (0.619-0.774)	0.762	0.674		0.560	0.669	0.705	0.724

^a^Average values are obtained from a total of 50 different analysis results.

^b^AUROC: are under the receiver operator characteristic.

^c^AUPRC: area under the precision-recall curve.

^d^GBM: gradient boosting machine.

^e^RF: random forest.

^f^SVM: support vector machine.

^g^XGB: eXtreme gradient boosting.

### Performance Evaluation of the OPTIVAN Algorithm

In subgroup analysis for the validation dataset, the AUROC values were 0.780 (95% CI 0.662-0.895) for patients with eGFR<60 mL/min/1.73 m^2^, 0.672 (0.549-0.795) for 60-89 mL/min/1.73 m^2^, and 0.725 (0.629-0.821) for ≥90 mL/min/1.73 m^2^. The AUROC for males was higher than that for females (0.791 vs 0.606). In the BMI subgroup analysis, the AUROC was 0.751 (95% CI 0.675-0.826) for the BMI 18.5-24.9 kg/m^2^ group and 0.621 (0.474-0.760) for the BMI 25.0-29.9 kg/m^2^ group (Table 4). The detailed comparison results between subgroups can be found in Figure S4 in Multimedia Appendix 1.

**Table 4:** Subgroup analyses of the performance of the OPTIVAN algorithm.

Subgroup				AUROC^a^ (95% CI)				
				Testing dataset^b^			Validation dataset^b^	
**eGFR^c^ (mL/min/1.73 m^2^)**								
	<60			0.667 (0.333-1.000)			0.780 (0.652-0.888)	
	60-89			0.700 (0.467-0.889)			0.672 (0.545-0.795)	
	≥90			0.896 (0.797-0.970)			0.725 (0.626-0.818)	
**Sex**								
	Male				0.885 (0.789-0.955)			0.791 (0.709-0.865)
	Female				0.855 (0.721-0.962)			0.606 (0.490-0.712)
**BMI (kg/m^2^)**								
		<18.5	0.886 (0.657-1.000)			0.644 (0.407-0.861)		
		18.5-24.9	0.859 (0.756-0.941)			0.751 (0.675-0.826)		
		25.0-29.9	0.845 (0.678-0.970)			0.621 (0.474-0.760)		
		≥30.0	0.834 (0.333-1.000)			0.630 (0.296-0.889)		

^a^AUROC: AUC of the receiver operator characteristic.

^b^Testing dataset refers to the results from an internal cohort, while validation dataset pertains to the results from an external cohort.

^c^eGFR: estimated glomerular filtration rate.

## Discussion

### Principal Results

We developed and evaluated the OPTIVAN algorithm, an ML-based tool designed to optimize initial vancomycin dosing using a minimal set of clinical variables. By incorporating key PK factors, this model enhances precision in dose selection for hospitalized adult patients, potentially improving therapeutic outcomes. A major strength of our study lies in the development of the algorithm using a large dataset and its external validation with independent hospital data. To develop the OPTIVAN algorithm, we selected 7 key features to predict whether AUC24/MIC of vancomycin would fall within the therapeutic range, accounting for individual patient PK variables before vancomycin administration. These features include age, BMI, glucose, BUN, eGFR (mL/min), hematocrit, and daily dose per body weight. Although ALT had a high importance score as a marker of disease severity, it was excluded from the final model due to its negative impact on predictive accuracy. Despite its association with disease severity, ALT’s high variability appeared to disrupt model stability, and its indirect relevance to vancomycin levels limited its utility in this context. Future analyses will consider comprehensive severity indices, such as the Acute Physiology and Chronic Health Evaluation II score, or alternative markers like albumin, to refine model performance as additional data become available.

Since vancomycin is mainly eliminated via renal excretion, its plasma concentration is highly dependent on renal function [29]. As renal function declines, the half-life of vancomycin increases and this leads to elevated blood concentration. Overall renal function is assessed by GFR and creatinine-based eGFR is the most commonly used measure in clinical practice [24,26]. It is well known that GFR physiologically declines with age [30]. BUN is an end product of protein metabolism, and tends to accumulate when renal function deteriorates [31]. In patients with diabetes, persistent hyperglycemia can lead to structural and functional damage to the renal vasculature and glomeruli, contributing to the development of diabetic nephropathy [32]. Given the strong dependence of vancomycin clearance on renal function, the inclusion of variables such as age, BMI, glucose, BUN, and eGFR in the OPTIVAN algorithm ensures robust predictive performance. Huang et al [33] conducted a study on vancomycin dose prediction using the XGB technique, in which composite variables incorporating low hematocrit and elevated creatinine concentrations exhibited high feature importance. There is minimal biological plausibility for hematocrit in the dose prediction model. One possible explanation is that it may serve as a surrogate for the volume of distribution, which is significantly increased in critically ill patients. However, the relationship between hematocrit and vancomycin metabolism remains unclear and requires further investigation. Notably, excluding hematocrit from the OPTIVAN algorithm resulted in a decline in its predictive performance, suggesting its potential role in dose optimization.

Vancomycin TDM using Bayesian modeling can provide dose prediction after 1-2 drug administrations and is known for its reasonably strong predictive validity [6]. In comparison, the primary advantage of the OPTIVAN algorithm developed in this study is its ability to predict the appropriate dose before administration. A key strength of the OPTIVAN algorithm is its reliance on variables commonly available in medical institutions, ensuring practical applicability. Demographic information and routine laboratory test results are accessible across all health care settings. Additionally, the ML-based TDM approach is more intuitive and easy to use in real-world clinical settings. Unlike Bayesian modeling, which requires specialized software and trained personnel, the OPTIVAN algorithm offers immediate dose predictions, making it more accessible to prescribing clinicians. While conventional TDM is typically conducted by clinical pathologists or pharmacists, a web-based ML algorithm allows physicians to directly assess whether their intended dose achieves therapeutic AUC24/MIC targets even without expertise in ML. This facilitates more efficient and timely vancomycin dose adjustments in clinical practice.

Using the SVM technique, the OPTIVAN algorithm demonstrated clinically acceptable performance, achieving the best AUROC of 0.832 for testing and 0.720 for external validation datasets. Additionally, it exhibited the highest average AUROC of 0.733 for testing and 0.720 for external validation datasets. The calibration plot of the SVM algorithm indicated minimal underestimation or overestimation. Based on the external validation results, the implementation of the OPTIVAN algorithm would likely predict approximate exposure in 72.0% (95% CI 65.4%-78.3%) of patients. There was variability in its predictive ability among subgroups, as it performed equally well among various renal function groups, (AUROC ranging from 0.672 to 0.780); however, there was greater variability among other patient characteristics including sex (0.791 for males and 0.606 for females) and BMI (ranging from 0.621 to 0.751). Further refinements may be necessary to improve generalization.

Typically, the results of conventional vancomycin TDM analysis can be determined after drug administration. In other words, the AUC24/MIC of vancomycin can be evaluated by measuring the drug concentrations after administration and subsequently conducting TDM analysis using Bayesian methods to assess the adequacy of the initial dosage. When analyzing the first requested vancomycin TDM after drug administration, the AUC24/MIC was within the therapeutic range in 37.1% of the internal cohort and 29.9% of the external cohorts. As mentioned earlier, while vancomycin dosing is recommended to be based on body weight and renal function, in real-world clinical practice, clinicians often rely on standard dosing based on their experience. Interestingly, our study found only a moderate to minimum correlation between daily dose per weight and BMI, suggesting that most patients were treated with standard doses of vancomycin rather than a weight-based dosing strategy. Since the OPTIVAN algorithm demonstrates a predictive performance of approximately 72.0% (95% CI 65.4%-78.3%) for vancomycin’s AUC24/MIC, using this algorithm in the initial drug dosage determination phase would make it over twice as likely for AUC24/MIC ratios to fall within the therapeutic range compared with decisions made solely by clinicians. These results support the notion that applying the OPTIVAN algorithm to the vancomycin treatment process in clinical practice can provide valuable assistance in medical decision-making.

### Comparisons With Prior Work

Previous studies have suggested vancomycin dosing nomograms based on body weight and renal function to reach a target trough concentration of 15-20 mg/L [34-36]. Other researchers have proposed recommended dosages by pharmacists for more efficient drug administration [37,38]. However, these approaches face limitations in addressing timely patient-specific clinical decisions. Recently, some groundbreaking studies have been introduced using various ML techniques for vancomycin TDM analysis [33,39]. However, some studies were either based on trough concentrations, which are not recommended in current vancomycin TDM guidelines [6], or focused exclusively on pediatric and neonatal populations [40,41]. Miyai et al [42] predicted the maintenance dose rather than the initial dose, lacked external validation, and did not specify model performance. The model by Bououda et al [43] focused on interdose rather than the initial dosing, relied on simulated rather than real patient data, and was validated using only 24 external cases. Furthermore, the lack of a web-based app in these studies restricts their practical clinical applicability.

### Enhance Clinical Utility

The web-based app using the OPTIVAN algorithm provides an easy-to-use interface for clinicians to input patient data and receive instant assessments of whether the predicted AUC24/MIC of vancomycin falls within the therapeutic range. The app is expected to improve vancomycin TDM efficiency by integrating into hospital or laboratory information systems. The OPTIVAN algorithm consists of 7 features. To improve user convenience, the web-based app includes 9 variables: sex, age, weight, height, creatinine, BUN, glucose, hematocrit, and planned daily dose. The eGFR, BMI, and daily dose per body weight can be automatically calculated using input variables. Upon inputting the data, the algorithm immediately predicts if the desired dosage falls within the therapeutic range.

### Limitations

Despite its robust validation, this study had limitations. First, the OPTIVAN algorithm was developed using retrospective data, which may introduce selection bias or data heterogeneity. To further refine the model, future research should focus on conducting prospective clinical trials to evaluate the algorithm’s real-world impact on vancomycin dosing accuracy and patient outcomes. Second, our model uses a binary classification approach, determining whether a given vancomycin dose is appropriate but does not provide a numerical AUC24/MIC value. Despite the substantial amount of data collected in our study, it appears that even more extensive data collection would be necessary to develop a robust regression model. However, we sought to address this limitation by providing users of the ML model with examples of appropriate initial drug doses from patients with similar conditions. This approach enables users to approximate the appropriate initial drug dose with nearly the same ease as a regression model. Finally, our model requires all input variable values to be present and does not accommodate missing data. Nevertheless, given that the input variables are typically collected in hospital settings, we believe the model can be effectively used in most patient cases.

### Conclusions

The OPTIVAN algorithm represents a significant advancement in ML-based precision dosing, addressing the limitations of empirical vancomycin dosing strategies. By leveraging readily available clinical data, this tool has the potential to improve initial dosing accuracy, minimize therapeutic failures, and reduce the burden of TDM adjustments, ultimately enhancing patient safety. By using a data-driven approach that considers individual patient characteristics, this algorithm could reduce the risks of treatment failure, complications, and drug-related adverse effects. The use of ML techniques to personalize initial vancomycin dosage is a significant advancement in treating MRSA infections, especially in regions with high prevalence.

## Appendix

Multimedia Appendix 1 Additional figures.

## Abbreviations

AUC24/MIC: area under the curve over 24 hours to minimum inhibitory concentration
AUPRC: AUC of the precision-recall curve
AUROC: AUC of the receiver operator characteristic
BUN: blood urea nitrogen
CKD-EPI: chronic kidney disease epidemiology collaboration
eGFR: estimated glomerular filtration rate
ML: machine learning
MRSA: methicillin-resistant Staphylococcus aureus
RF: random forest
SVM: support vector machine
TDM: therapeutic drug monitoring
XGB: eXtreme gradient boosting

## Data Availability

The datasets used in this study are not publicly available due to the inclusion of patient’s clinical information, but may be available from the corresponding author on reasonable request depending on the policy and procedures of the institutions.
