# Supplementary material for: Optimizing Initial Vancomycin Dosing in Hospitalized Patients Using Machine Learning Approach for Enhanced Therapeutic Outcomes: Algorithm Development and Validation Study
Source: J Med Internet Res. 2025 Mar 31;27:e63983. doi: 10.2196/63983 (PMC11997519; doi:10.2196/63983)
Supplement: Multimedia Appendix 1 [file jmir_v27i1e63983_app1.docx]

**Multimedia Appendix 1**


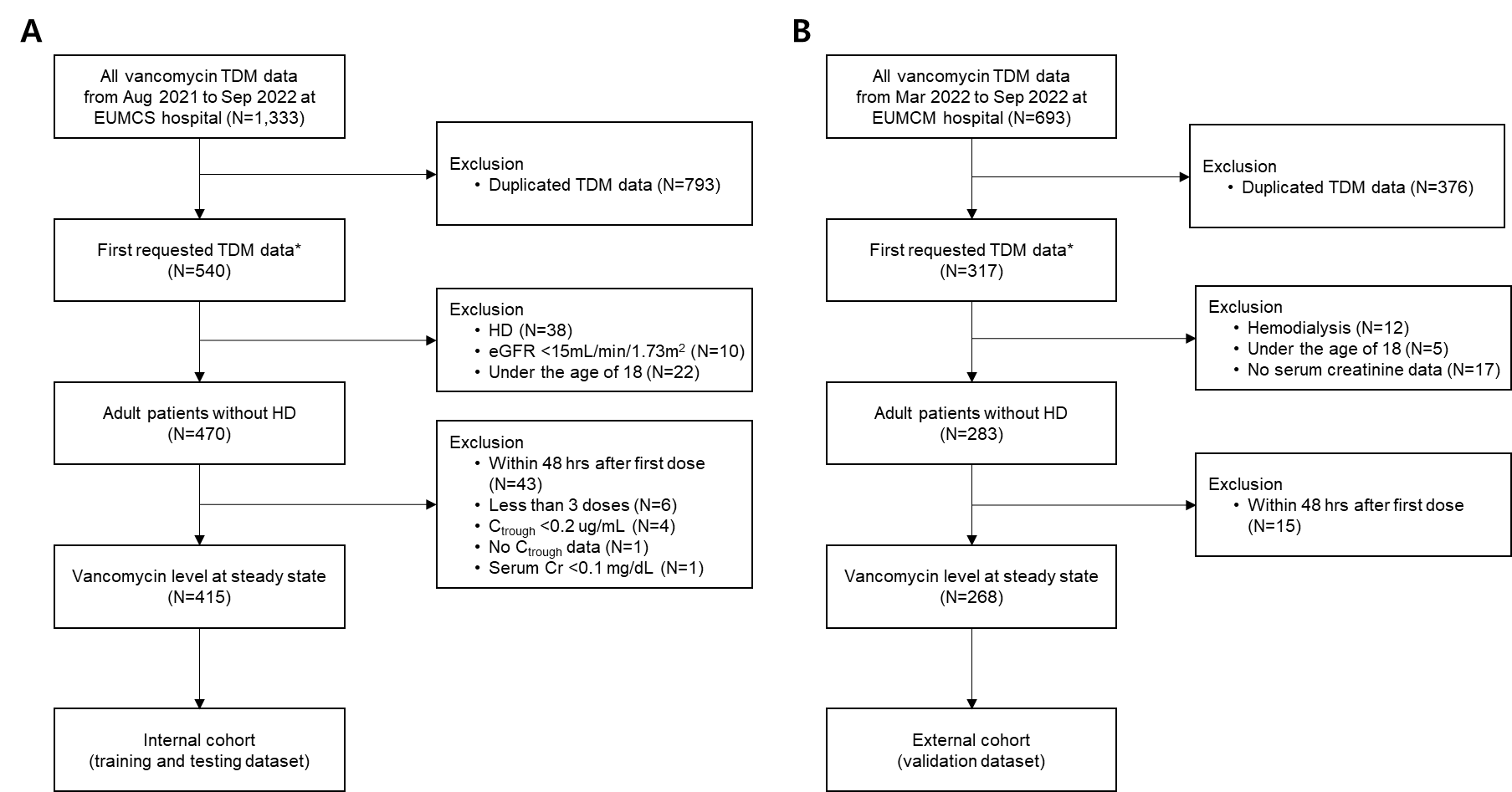


**Figure S1. Flow chart of the study subject selection process**. (A) Development dataset for training and testing machine learning algorithms and (B) external validation dataset.

*If multiple requests for vancomycin TDM were received from the same patient, we selected the first set of TDM data.

Abbreviations: Cr, creatinine; C_trough_, trough concentration of vancomycin; eGFR, estimated glomerular filtration rate; EUMCM, Ewha Womans University Mokdong; EUMCS, Ewha Womnans University Seoul; HD, hemodialysis; TDM, therapeutic drug monitoring

**Figure S2.** **Analysis of correlation between features**. No significant correlations were found among the finally selected variables including age, estimated glomerular filtration rate (eGFR, mL/min), daily dose per weight (D_dailydose_perwt), glucose, blood urea nitrogen (BUN), hematocrit (Hct), and body mass index (BMI).


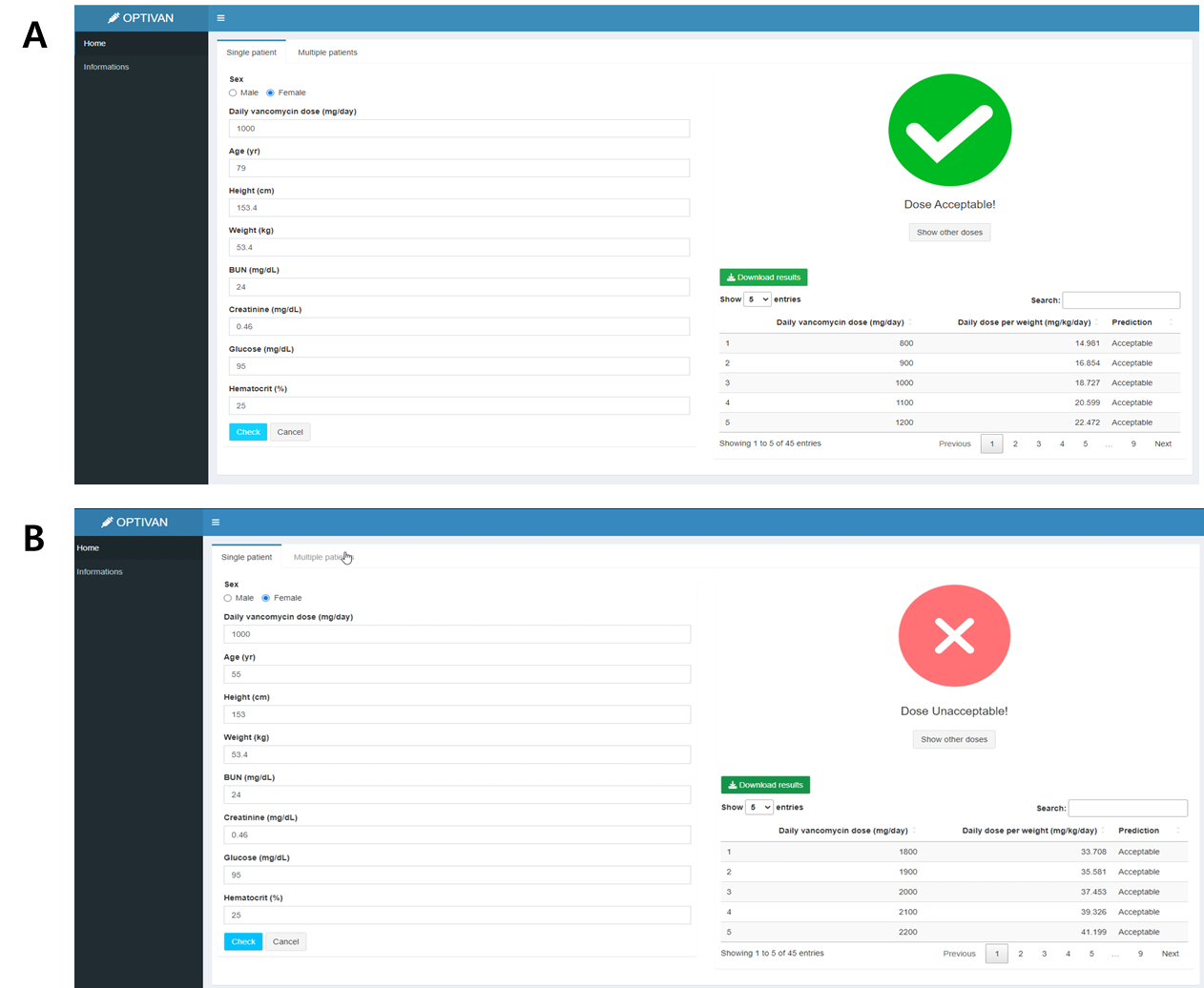


**Figure S3. Screenshot of the Web application.** Examples of a drug dosage within the therapeutic range (A) and exceeding the therapeutic range (B).


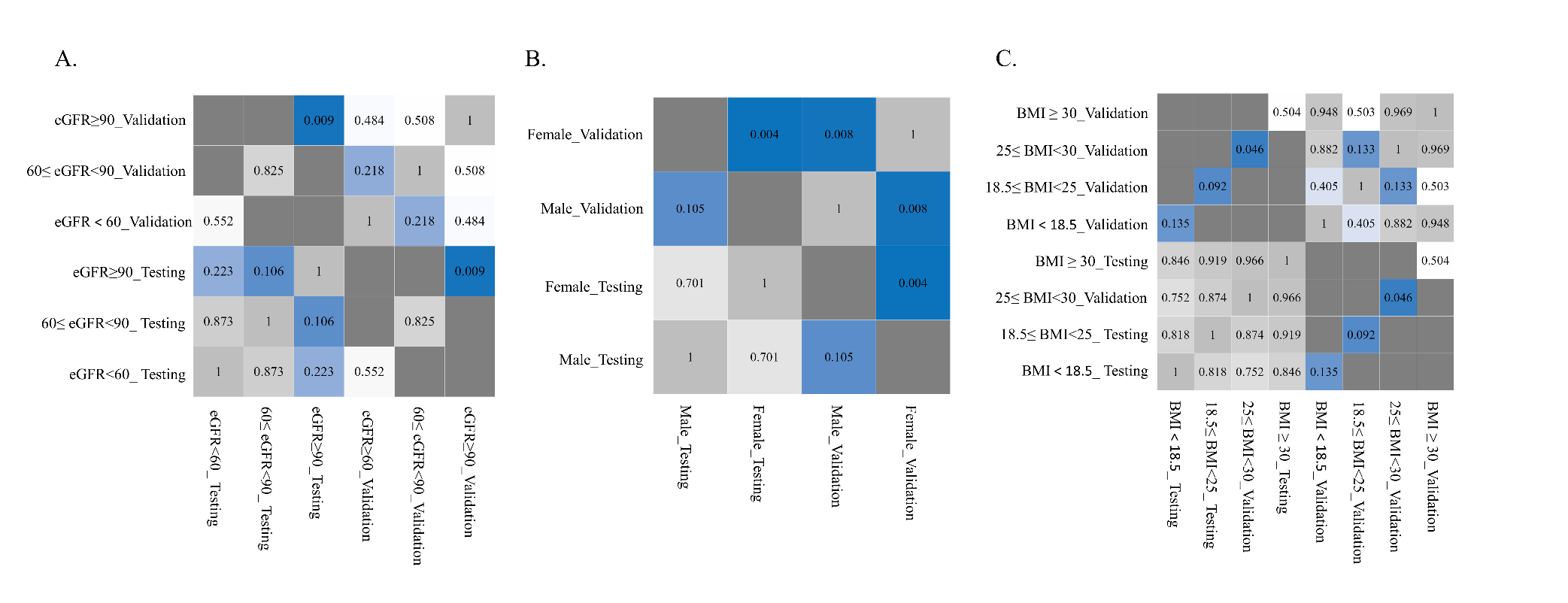


**Figure S4**. **Results of DeLong test between each group**. (A) eGFR, (B) gender, and (C) BMI. To compare the validation result of each subgroup and data set, examined using DeLong test and got a p-value. Dark gray regions, which are no value, means these groups aren’t compared.

Abbreviations: BMI, body mass index (kg/m^2^); eGFR, estimated glomerular filtration rate (mL/min/1.73m^2^).
